# Risk factors for mortality among malnourished HIV-infected adults eligible for antiretroviral therapy

**DOI:** 10.1186/s12879-016-1894-3

**Published:** 2016-10-12

**Authors:** Susannah L Woodd, Paul Kelly, John R. Koethe, George Praygod, Andrea M. Rehman, Molly Chisenga, Joshua Siame, Douglas C. Heimburger, Henrik Friis, Suzanne Filteau

**Affiliations:** 1 Faculty of Epidemiology and Population Health, London School of Hygiene & Tropical Medicine, Keppel Street, London, WC1E 7HT UK; 2 Barts & the London School of Medicine and Queen Mary University of London, London, UK; 3 Vanderbilt University, Nashville, USA; 4 National Institute for Medical Research, Mwanza, Tanzania; 5 University Teaching Hospital, Lusaka, Zambia; 6 University Teaching Hospital, Lusaka, Zambia; 7 University of Copenhagen, Copenhagen, Denmark

**Keywords:** Mortality risk factors, Malnutrition, ART, Africa

## Abstract

**Background:**

A substantial proportion of HIV-infected adults starting antiretroviral therapy (ART) in sub-Saharan Africa are malnourished. We aimed to increase understanding of the factors affecting their high mortality, particularly in the high-risk period before ART initiation.

**Methods:**

We analysed potential risk factors for mortality of Zambian and Tanzanian participants enrolled in the NUSTART clinical trial. Malnourished adults (*n =* 1815; body mass index [BMI] <18.5 kg/m^2^) were recruited at referral to ART and randomised to receive different nutritional supplements. Demographics, measures of body composition, blood electrolytes and clinical conditions were investigated as potential risk factors using Poisson regression models.

**Results:**

The mortality rate was higher in the period from referral to starting ART (121 deaths/100 person-years; 95 % CI 103, 142) than during the first 12 weeks of ART (66; 95 % CI 57, 76) and was not affected by trial study arm. In adjusted analyses, lower CD4 count, BMI and mid-arm circumference and raised C-reactive protein were associated with an increased risk of mortality throughout the study. Male sex and lower hand-grip strength carried an increased risk in the pre-ART period. Participants on tuberculosis treatment at referral had a lower mortality rate (adjusted Rate Ratio 0.44; 95 % CI 0.31, 0.63).

**Conclusion:**

Among malnourished ART-eligible adults, pre-ART mortality was twice that in the early post-ART period, suggesting many early ART deaths represent advanced HIV disease rather than treatment-related events. Therefore, more efforts are needed to promote earlier diagnosis and immediate initiation of ART, as recently recommended by WHO for all persons with HIV worldwide. The positive effect of tuberculosis treatment suggests undiagnosed tuberculosis is a contributor to mortality in this population.

**Trial registration:**

Pan African Clinical Trials Registry, PACTR201106000300631; registered on 1st June 2011.

**Electronic supplementary material:**

The online version of this article (doi:10.1186/s12879-016-1894-3) contains supplementary material, which is available to authorized users.

## Background

The last decade has seen great advances in expanding access to antiretroviral therapy (ART) for HIV-infected Africans. However, high mortality immediately before and in the first few months after starting ART remains a major concern [1, 2]. Consistent risk factors for early mortality include delayed ART initiation, indicated by very low CD4 count or advanced WHO disease stage, the presence of opportunistic infections such as tuberculosis (TB), and low body mass index (BMI) [1–3]. Male sex, low haemoglobin, low serum phosphate and older or younger age are additional risk factors for mortality in some cohorts [4–9].

In our recent Nutritional Support for African Adults Starting Antiretroviral Therapy (NUSTART) trial we addressed malnutrition as a risk factor for mortality by randomizing underweight African adults initiating ART to a lipid-based nutritional supplement (LNS) containing multiple vitamins and minerals compared to LNS alone. While we observed no difference in mortality between treatment arms, the trial design provided a unique opportunity for detailed clinical assessments of malnourished, HIV-infected persons both prior to ART-initiation and during the early months of treatment [10, 11]. We noted that the mortality rate between referral for ART and 12 weeks after starting ART was considerably higher, 83/100-person-years (95 % confidence interval (CI) 75, 92), than in most other reports of early mortality in patients starting ART in low-income settings [1–3, 6, 12].

Many studies have reported mortality risk factors among patients starting ART but, as far as we know, none has included the high-risk period between referral for ART and actually starting ART in a large cohort of malnourished patients. Given the very high mortality observed, we felt it important to analyse further the extensive NUSTART database, including clinical conditions, blood electrolytes and body composition, to determine risk factors that could be used to improve clinical understanding and management of this vulnerable population.

## Methods

### Design and intervention

The NUSTART trial was a randomised, controlled phase III trial with the primary outcome of mortality from recruitment at referral to ART until 12 weeks after starting ART and registered as PACTR201106000300631; details have been described previously [10]. Briefly, the study was conducted between August 2011 and December 2013 at two sites: the National Institute for Medical Research (NIMR), Mwanza, Tanzania and the University Teaching Hospital (UTH), Lusaka, Zambia.

The intervention was designed to mimic protocols for nutritional management of severely malnourished children [13] and was a lipid-based nutritional supplement, made for the trial by Nutriset, Malaunay, France, either with high levels of vitamins and minerals (LNS-VM) or without (LNS). From recruitment at referral for ART to 2 weeks after starting ART, the LNS-VM or LNS was given with minimal calories, i.e. 30 g/day, ~150 kcal/day. From 2 to 6 weeks after initiating ART the LNS-VM or LNS were given in 250 g/day, comprising ~1400 kcal/day. Patients were followed to 12 weeks of ART.

### Participant recruitment and follow-up

Adults attending free HIV testing services at both sites, eligible for ART and fulfilling the study inclusion criteria were deemed eligible for the trial. Inclusion criteria were: at least 18 years old, ART-naive (except for previous short-term regimens to prevent maternal-to-child HIV transmission), BMI < 18.5 kg/m^2^, requiring ART as determined by CD4 count <350 cells/μl or stage 3 or 4 disease, willing to undertake intensive follow-up in the study clinic and providing written (or thumbprint) informed consent. Exclusion criteria were participation in a potentially conflicting research protocol, or pregnancy by self-report.

Medical care, including ART, was provided primarily by local health services though study staff treated minor illnesses and referred as necessary during patients’ study visits. National treatment guidelines for ART regimen differed in the two countries: 74 % Zambians were prescribed efavirenz, tenofovir, and emtricitabine, whereas in Tanzania a wider range of regimens was used (all comprising two nucleoside/nucleotide reverse transcriptase inhibitors and a non-nucleoside reverse transcriptase inhibitor).

Patients were seen weekly from recruitment until they initiated ART and then at 2, 4, 6, 8, and 12 weeks after starting ART. Patients who were ill were invited to come for unscheduled visits at any time. Patients who missed scheduled visits were actively followed up through phone-calls to patients and relatives, contact with their ART clinic and additionally in Mwanza by visiting patients at home. We therefore ascertained the primary outcome of mortality before 12 weeks ART in 91 % of the 1815 participants.

### Data collection

#### Anthropometry, body composition, grip strength, and haemoglobin

Patient weight and mid-upper arm circumference were measured at all scheduled visits, and height at recruitment. Measurements were taken in triplicate and the median used in analyses. Staff were trained according to standard protocols [14]. Technical errors of measurement were within acceptable limits [15]. Body fat and lean proportions were measured by bioelectrical impedance (Tanita BC-418, Tokyo, Japan) and calibrated against air displacement plethysmography results from a sample of Lusaka patients [16]. Values were divided by patient’s height squared to create an index. Grip strength was measured using a digital handgrip dynamometer (Takei TKK 5401, Chasmor, UK); two measurements were taken from each hand and the machine takes the average of the two maximum readings. Haemoglobin was measured in the clinic by Hemocue (Angelholm, Sweden).

#### Laboratory analyses

CD4 count was measured by local central clinical services. Serum C-reactive protein (CRP) was measured by ELISA (Assaypro, St Charles, MO, USA) in the site laboratories. At both sites values for external quality control (QC) sera (Assaypro) were within expected ranges but both the sites had high inter-assay coefficients of variation (CV): 32 % in Lusaka and 37 % in Mwanza. In Lusaka, serum phosphate was measured on a Pointe 180 analyser (Pointe Scientific, Ann Arbor, MI, USA). Sample results were only accepted from runs for which the external QC was within the expected range. Inter-assay CV was 7 % for this external QC. In Mwanza, serum phosphate was measured by Roche COBAS Integra 400 analyser in an external laboratory (Bugando Medical Centre).

#### Appetite

Appetite was measured by a 4-question questionnaire, combined to produce an appetite score using polychoric correlation [17]. Lower scores represent lower appetite. Preliminary analysis [18] indicated that the test results were clustered in Lusaka and the score correlated best with subsequent weight change in Tanzanian patients. Therefore effect of appetite was only analysed for Tanzanian patients.

#### Clinical signs and treatment

At each visit, patients were asked if they were taking medications, including anti-TB drugs and cotrimoxazole. All patients were examined at recruitment and assessed for signs of oedema. When a patient died in hospital the diagnosis recorded in the hospital records was taken as the cause of death. Many patients died at home and the cause of death was considered to be unknown.

### Sample size

The planned sample size of 2300 participants was based on the primary outcome of mortality. Recruitment was stopped early because of a higher than expected mortality rate as previously described [10]. The final sample size of 1815 was sufficient to detect, at 90 % power, a mortality difference of 30 % between trial arms.

### Data management and statistics

Data were double entered, checked and analyses were conducted in STATA version 13.1. Risk factors at baseline which were analysed for their associations with mortality during the study were sex, age, socioeconomic status (SES), CD4 count, BMI, haemoglobin, mid-upper arm circumference (MUAC), fat-free mass index, fat-mass index, oedema, TB treatment, cotrimoxazole treatment, serum phosphate and CRP, grip strength, and appetite score (for Tanzanians only). Principal components analysis (PCA), used to describe the SES of the population, [19] was conducted separately for each country since there were notable differences between sites; variables offered into the PCA were housing characteristics, sanitation, water source, and ownership of electrical goods, animals and modes of transport.

BMI, haemoglobin, fat-free mass index, fat mass index and grip strength were used as continuous variables in regression analysis, after testing for linearity using fractional polynomials. Age, CD4 count and CRP did not have linear relationships and were categorised using standard cut-offs for CD4 and in 4 groups for age (18–29, 30–39, 40–49, ≥50) and CRP (<10 mg/l, 10–49 mg/l, 50–159 mg/l, ≥160 mg/l including the maximum cut-off for most plates of 160 mg/l). Appetite score was divided into tertiles. Serum phosphate was analysed as restricted cubic splines with 4 knots to reflect the increased mortality at high and low values. Low values were defined by US National Institutes of Health Division of AIDS criteria [20] and high values were considered any above the normal range [21]. Continuous variables were also categorised according to standard cut-offs or tertiles for presentation of mortality rates.

Poisson regression was used to estimate hazard ratios. Follow-up time was censored at 10 weeks if patients had not yet started ART (*n =* 115) and these patients were excluded from all post-ART analysis to avoid the distorting effect of a prolonged time from risk-factor measurement to post-ART period. Factors associated with mortality in univariable analysis were investigated in multivariable models controlling for age, sex, CD4 count, BMI, TB treatment, oedema, grip strength, treatment arm, study site and follow-up time. BMI was omitted from the model when analysing fat-free mass index and MUAC due to collinearity and when analysing appetite as it could be considered a mediator. Statistical significance was assessed using Wald tests. After analysis of the total follow-up time, the study was split into pre- and post-ART periods and baseline factors assessed for association with mortality in each period. Thirty-one patients were excluded due to missing information on ART start date, including 19 patients who died. Interactions between each risk factor and study period were tested in univariable analysis. Length of time spent pre-ART was controlled for in post-ART analyses.

## Results

Table 1 describes the population at recruitment. Characteristics were similar at baseline between treatment arms [10]. Half the cohort was male. At baseline a quarter of participants had CD4 count < 50 cells/μl and mean BMI was 16.7 kg/m^2^. Two-thirds of participants had moderate or severe anaemia, 11 % had low serum phosphate and 17 % reported taking TB treatment at their recruitment visit (Table 1).

**Table 1:** Baseline characteristics of the study population^a^

Variable	Level	*n* (%)
*N* (%)		1815 (100)
Lusaka		1111 (61.2)
Age (years), median (IQR)		35 (29–41)
Female		900 (49.6)
BMI (kg/m^2^), median (IQR)		16.7 (15.6–17.5)
BMI <17 kg/m^2^		1074 (59.2)
CD4 count (cells/μl), median (IQR)		120 (51–211)
CD4 count group	> = 200	494 (27.2)
	100–199	531 (29.3)
	50–99	347 (19.1)
	<50	443 (24.4)
Haemoglobin (g/L), median (IQR)		96 (80–111)
Haemoglobin group^b^	Normal	177 (9.8)
	Mild anaemia	285 (15.7)
	Moderate anaemia	810 (44.6)
	Severe anaemia	398 (21.9)
	Missing	145 (8.0)
Phosphate <0.87 mmol/L	<0.87	196 (10.8)
	Missing	51 (2.8)
Marital status	Married/Cohabiting	858 (47.3)
	Widowed	203 (11.2)
	Divorced-Separated	510 (28.1)
	Single	243 (13.4)
	Missing	1 (<0.1)
Occupation	Salaried	271 (14.9)
	Self-employed	945 (52.1)
	Housewife	183 (10.1)
	Student	18 (1.0)
	Unemployed	397 (21.9)
	Missing	1 (<0.1)
Education	None	343 (18.9)
	Primary	1045 (57.6)
	Secondary	377 (20.8)
	Tertiary	49 (2.7)
	Missing	1 (<0.1)
TB treatment at baseline		304 (16.9)
Oedema at baseline		66 (3.6)
Initial ART regimen (% of 1474 patients who started ART)	AZT/3TC/EFV	135 (9.2)
	AZT/FTC/NVP	236 (16.0)
	TDF/FTC/EFV	816 (55.4)
	TDF/FTC/NVP	64 (4.3)
	Other	54 (3.7)
	Missing	169 (11.5)

^a^ *BMI* body mass index, *CI* confidence interval, *LNS* lipid-based nutritional supplement without added vitamins and minerals, *LNS-VM* lipid nutritional supplement with added vitamins and minerals, *AZT* Zidovudine, *3TC* Lamivudine, *NVP* nevirapine, *TDF* tenofovir, *EFV* efavirenz; *FTC* emtricitabine

^b^Haemoglobin categories were selected based on usual nutritional cut-offs, not as defined for specific adverse events; adequate haemoglobin was defined as 130 g/L for men and 120 g/L for women; mild anaemia was 110 g/L to the adequate cut-off; moderate anaemia was 80–109 g/L; severe anaemia was < 80 g/L

Participants spent a median of 14.3 weeks in the study (IQR 10.4 to 15.7) and a median of 3 weeks (IQR 2.1 to 4.7) in the pre-ART period. The mortality rate from recruitment until 12 weeks after starting ART was 83.1 deaths/100 person-years (95 % CI 75.0 to 92.1). In the pre-ART period it was particularly high at 120.8 (95 % CI 103.0 to 141.7) and the rate increased as participants spent longer before starting ART, whereas it dropped to 66.1 (95 % CI 57.4 to 76.1) after ART initiation (Table 2).

**Table 2:** Mortality rates by potential baseline risk factors^a^

		*n*	deaths	Mortality rate per 100 person-years (95 % CI)
Total population		1815	365	83.1 (75.0 to 92.1)
Study period	Pre-ART	1784^e^	151	120.8 (103.0 to 141.7)
	Post-ART	1441	195	66.1 (57.4 to 76.1)
Days Pre-ART	0–13	1784	46	73.6 (55.1 to 98.3)
	14–20	1401	25	119.0 (80.4 to 176.2)
	21–27	867	20	151.4 (97.6 to 234.6)
	28–70	554	60	211.9 (164.5 to 272. 9)
Days Post-ART	0–13	1441	44	81.3 (60.5 to 109.2)
	14–27	1380	39	75.5 (55.1 to 103.3)
	28–41	1326	35	70.4 (50.5 to 98.0)
	42–55	1277	32	66.9 (47.3 to 94.6)
	56–69	1266	24	52.2 (35.0 to 77.9)
	≥70	1184	21	46.0 (30.0 to 70.5)
Site	Lusaka	1111	170	64.4 (55.4 to 74.9)
	Mwanza	704	195	122.3 (106.3 to 140.8)
Sex	Women	900	156	72.6 (62.0 to 84.9)
	Men	915	209	100.3 (87.6 to 114.9)
Age group (years)	18–29	463	85	80.1 (64.7 to 99.1)
	30–39	788	159	87.5 (74.9 to 102.2)
	40–49	411	81	82.2 (66.1 to 102.1)
	> = 50	153	40	108.3 (79.4 to 147.6)
Socioeconomic quintiles	Highest	360	73	85.4 (67.9 to 107.5)
	High	355	77	93.0 (74.3 to 116.2)
	Middle	362	72	84.7 (67.2 to 106.7)
	Low	374	81	93.9 (75.5 to 116.8)
	Lowest	364	62	74.0 (57.7 to 94.9)
CD4 group (cells/μl)	> = 200	494	59	48.1 (37.3 to 62.1)
	100–199	531	82	64.5 (51.9 to 80.1)
	50–99	347	88	112.0 (90.9 to 138.0)
	<50	443	136	143.3 (121.1 to 169.5)
BMI group (kg/m^2^)	17.0–18.49	741	106	57.7 (47.7 to 69.8)
	16.0–16.9	468	93	85.5 (69.8 to 104.8)
	<16.0	606	166	126.9 (109.0 to 147.7)
Haemoglobin (g/L) ^b^	> = 120/130	177	15	33.2 (20.0 to 55.0)
	110–119/129	285	39	53.7 (39.2 to 73.4)
	80–190	810	158	83.5 (71.4 to 97.6)
	<80	398	127	153.9 (129.3 to 183.1)
Oedema	No	1749	335	81.5 (73.2 to 90.7)
	Yes	66	30	245.5 (171.7 to 351.1)
On Tuberculosis Treatment	No	1511	320	93.0 (83.3 to 103.7)
	Yes	304	38	50.2 (36.5 to 69.0)
On cotrimoxazole	No	319	59	77.7 (60.2 to 100.3)
	Yes	1486	302	87.5 (78.2 to 98.0)
Serum phosphate^c^	Grades 3–4 low, <0.65 mmol/L	54	16	134.0 (82.1 to 218.8)
	Grades 1–2 low, 0.65–0.87 mmol/L	142	28	83.5 (57.7 to 120.9)
	Normal, 0.87–1.45 mmol/L	1241	232	79.6 (70.0 to 90.5)
	Above normal, >1.45 mmol/L	327	74	98.3 (78.3 to 123.5)
CRP group (mg/l)	<10	358	27	28.7 (19.7 to 41.8)
	10–49	450	57	50.7 (39.1 to 65.8)
	50–159	491	118	109.2 (91.1 to 130.7)
	≥160	463	151	155.8 (132.8 to 182.7)
Fat-free mass index^c^ Tertiles (kg/m^2^)	≥14.5	486	65	55.6 (43.6 to 70.9)
	13.7 to 14.49	503	81	65.2 (52.4 to 81.0)
	<13.7	471	92	81.4 (66.4 to 99.9)
Fat mass index^c^ Tertiles (kg/m^2^)	≥3.0	466	62	52.2 (40.7 to 67.0)
	2.2 to 2.99	520	80	62.3 (50.0 to 77.5)
	<2.2	474	96	89.8 (73.5 to 109.7)
Grip strength^c^ Tertiles (kg)	>22.2	592	79	53.6 (43.0 to 66.8)
	16.2 to 22.2	588	119	85.9 (71.8 to 102.8)
	<16.2	572	155	125.7 (107.4 to 147.1)
MUAC^c^ Tertiles (cm)	>22.3	603	63	41.4 (32.3 to 52.9)
	20.5–22.3	633	123	82.8 (69.4 to 98.8)
	<20.5	566	179	150.3 (129.8 to 174.0)
Appetite^c, d^ Tertiles	> − 0.5	229	48	86.5 (65.2 to 114.8)
	−1.48 to −0.55	227	59	112.8 (87.4 to 145.6)
	<−1.5	228	76	158.1 (126.3 to 197.9)

^a^Abbreviations: *BMI* body mass index, *MUAC* mid-upper arm circumference, *CRP* C-reactive protein

^b^Normal haemoglobin cut-offs used were 120 g/L for women and 130 g/L for men

^c^Values were divided into tertiles for factors without established cut-offs

^d^Appetite score from polychoric correlation

^e^31 patients excluded due to missing information on ART

Higher mortality rates were observed in participants who were male, Tanzanian, or at recruitment had very low CD4, BMI, haemoglobin, grip strength or appetite scores, had very high CRP, had severely low or high phosphate or oedema (Table 2). Grip strength and MUAC showed strong independent associations with mortality; rate of death decreased 6 % for every 1 kg increase in grip strength and 13 % for every 1 cm gain in MUAC. Patients receiving TB treatment at baseline had less than half the mortality rate of those not on treatment, after adjusting for other factors including CD4 count. Age, SES and treatment with co-trimoxazole at recruitment had no association with mortality. Appetite score, phosphate and fat-mass index showed crude associations but these did not persist after adjusting for other factors (Table 3).

**Table 3:** Poisson regression models for baseline factors associated with overall mortality

Factor	Level	Unadjusted HR (95 % CI) ^b^	*p*-value	Adjusted HR (95 % CI) ^c^ *n =* 1685^c^	Adjusted *p*-value
Site	Zambia	1	<0.001	1	<0.001
	Tanzania	1.90 (1.55 to 2.33)		1.60 (1.27 to 2.03)	
Sex	Female	1	0.002	1	<0.001
	Male	1.38 (1.12 to 1.70)		2.05 (1.60 to 2.64)	
Age group (years)	18–29	1	0.43	1	0.79
	30–39	1.09 (0.84 to 1.42)		1.01 (0.77 to 1.34)	
	40–49	1.03 (0.76 to 1.39)		0.94 (0.67 to 1.30)	
	> = 50	1.35 (0.93 to 1.97)		0.84 (0.55 to 1.28)	
Socioeconomic quintiles	Highest	1	0.65		
	High	1.09 (0.79 to 1.50)			
	Middle	0.99 (0.72 to 1.37)			
	Low	1.10 (0.80 to 1.51)			
	Lowest	0.87 (0.62 to 1.22)			
CD4 group (cells/μl)	> = 200	1	<0.001	1	<0.001
	100–199	1.34 (0.96 to 1.87)		1.19 (0.84 to 1.69)	
	50–99	2.33 (1.67 to 3.24)		1.69 (1.19 to 2.40)	
	<50	2.98 (2.19 to 4.04)		2.10 (1.52 to 2.91)	
BMI^a^	Per 1 kg/m^2^ increase	0.76 (0.71 to 0.81)	<0.001	0.85 (0.78 to 0.91)	<0.001
Haemoglobin	Per 10 g/L increase	0.82 (0.78 to 0.86)	<0.001		
Oedema	No	1	<0.001	1	0.004
	Yes	3.01 (2.07 to 4.38)		1.84 (1.21 to 2.78)	
Baseline TB^a^ treatment	No	1	<0.001	1	<0.001
	Yes	0.54 (0.39 to 0.76)		0.44 (0.31 to 0.63)	
Baseline cotrimoxazole treatment	No	1	0.41		
	Yes	1.13 (0.85 to 1.49)			
CRP^a^ (mg/l)	<10	1	<0.001	1	<0.001
	10–49	1.77 (1.12 to 2.80)		1.61 (1.00 to 2.58)	
	50–159	3.80 (2.50 to 5.78)		3.03 (1.97 to 4.66)	
	≥160	5.43 (3.61 to 8.18)		3.67 (2.40 to 5.62)	
Fat-free mass index	Per 1 kg/m^2^ increase	0.76 (0.66 to 0.88)	<0.001	0.80 (0.67 to 0.95)	0.01
Fat mass index	Per 1 kg/m^2^ increase	0.75 (0.64 to 0.88)	0.001	0.86 (0.70 to 1.05)	0.15
Grip strength	Per 1 kg increase	0.94 (0.93 to 0.96)	<0.001	0.94 (0.92 to 0.96)	<0.001
MUAC^a^	Per 1 cm increase	0.78 (0.74 to 0.82)	<0.001	0.87 (0.82 to 0.92)	<0.001
Appetite score	> − 0.5	1	0.004	1	0.65
	−1.49 to−0.55	1.30 (0.89 to 1.91)		1.20 (0.81 to 1.78)	
	<−1.5	1.83 (1.27 to 2.62		1.08 (0.72 to 1.61)	
Phosphate (mmol/l)	Cubic spline		0.001		0.40

^a^Abbreviations: *BMI* body mass index, *MUAC* mid-upper arm circumference, *CRP* C-reactive protein, *TB* tuberculosis

^b^ *n =* 1815 except haemoglobin *n =* 1670, CRP *n =* 1762, fat-free mass *n =* 1461, grip strength *n =* 1752, MUAC *n =* 1802, appetite *n =* 684 (Tanzanian patients only), phosphate *n =* 1764

^c^Multivariable analysis adjusted for age, sex, CD4, BMI, oedema, TB treatment, CRP, grip strength, site, follow-up time in 3 week bands (association with mortality *p <* 0.002 except in appetite analysis *p =* 0.24) and study arm. Association with fat-free mass, MUAC and appetite not adjusted for BMI. *n =* 1685 except fat-free mass *n =* 1349, MUAC *n =* 1672, appetite *n =* 630, (Tanzanian patients only), Phosphate *n =* 1644

In unadjusted analysis there is good evidence that the association between CRP and mortality changes with study period (*p =* 0.01) and in adjusted analysis the effect of CRP on mortality appears larger after starting ART than before (Table 4). There is at least weak evidence of interactions between study period and each of study site, sex and grip strength. In each case there is a strong association with mortality in the pre-ART period with Tanzanians, men and those with low grip strength having an increased mortality rate. However, these association do not appear to persist after starting ART. BMI, fat-free mass index and MUAC measured at recruitment have similar hazard ratios before and after starting ART and no evidence of interaction with study period.

**Table 4:** Poisson regression models for baseline factors associated with mortality during the pre- and post-ART periods^a^

		Pre-ART period (*n =* 1656) ^c^		After starting ART (*n =* 1334) ^d^	
Factor	Level	Adjusted HR^b^ (95 % CI)	*p*-value	Adjusted HR^b^ (95 % CI)	*p*-value
Site	Zambia	1	<0.001	1	0.28
	Tanzania	2.65 (1.82 to 3.84)		1.20 (0.86 to 1.66)	
Sex	Female	1	<0.001	1	0.16
	Male	3.44 (2.31 to 5.14)		1.29 (0.90 to 1.84)	
Age group (years)	18–29	1	0.65	1	0.71
	30–39	1.18 (0.77 to 1.83)		0.81 (0.55 to 1.20)	
	40–49	1.04 (0.62 to 1.75)		0.87 (0.56 to 1.35)	
	> = 50	0.83 (0.43 to 1.60)		1.00 (0.56 to 1.76)	
CD4 group (cells/μl)	> = 200	1	0.004	1	0.001
	100–199	1.23 (0.69 to 2.19)		1.34 (0.83 to 2.14)	
	50–99	2.08 (1.21 to 3.59)		1.60 (0.97 to 2.64)	
	<50	2.25 (1.35 to 3.59)		2.33 (1.49 to 3.66)	
BMI^a^	Per 1 kg/m^2^ increase	0.93 (0.82 to 1.05)	0.23	0.80 (0.72 to 0.89)	<0.001
Oedema	No	1	0.13	1	0.03
	Yes	1.57 (0.88 to 2.82)		2.03 (1.09 to 3.79)	
Baseline TB^a^ treatment	No	1	<0.001	1	0.003
	Yes	0.32 (0.18 to 0.58)		0.49 (0.30 to 0.79)	
CRP^a^ (mg/l)	<10	1	0.001	1	<0.001
	10–49	0.69 (0.32 to 1.49)		3.00 (1.49 to 6.05)	
	50–159	2.02 (1.13 to 3.62)		4.79 (2.43 to 9.43)	
	≥160	1.94 (1.09 to 3.45)		5.94 (3.02 to 11.67)	
Fat-free mass index	Per 1 kg/m^2^ increase	0.97 (0.74 to 1.26)	0.78	0.70 (0.56 to 0.89)	0.003
Grip strength	Per 1 kg increase	0.90 (0.87 to 0.93)	<0.001	0.98 (0.95 to 1.01)	0.12
MUAC^a^	Per 1 cm increase	0.93 (0.85 to 1.02)	0.11	0.84 (0.78 to 0.91)	<0.001
Appetite score	> − 0.5	1	0.34	1	0.85
	−1.49 to−0.55	0.90 (0.50 to 1.61)		1.00 (0.54 to 1.82)	
	<−1.5	1.31 (0.76 to 2.28)		1.15 (0.65 to 2.05)	
Phosphate (mmol/l)	Cubic spline		0.06		0.50

^a^Abbreviations: *ART* antiretroviral therapy, *BMI* body mass index, *MUAC* mid-upper arm circumference, *CRP* C-reactive protein, *TB* tuberculosis

^b^Multivariable analysis adjusted for age, sex, CD4, BMI, oedema, TB treatment, CRP, grip strength, site, follow-up period and study arm. Post-ART analysis also adjusted for weeks spent pre-ART. Association with fat-free mass, MUAC and appetite not adjusted for BMI. Post-ART analysis also adjusted for weeks spent pre-ART

^c^Pre-ART analysis; Fat-free mass *n =* 1331, MUAC *n =* 1643, Appetite *n =* 623, Phosphate *n =* 1615

^d^Post-ART analysis; Fat-free mass *n =* 1113, MUAC *n =* 1323, Appetite *n =* 478, Phosphate *n =* 1304

Baseline TB treatment was protective against mortality in both time periods. A secondary analysis of 1190 patients not on TB treatment at recruitment also shows a lower post-ART mortality rate among 117 patients who started TB treatment prior to initiating ART (HR 0.37 95 % CI 0.19, 0.72 *p*-value 0.004).

**Fig. 1:**
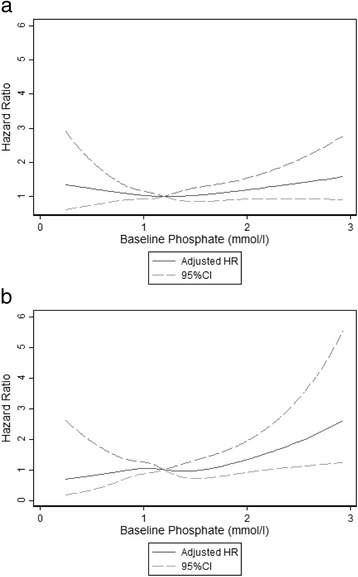
Adjusted hazard ratio* for baseline phosphate compared to median value of 1.2 mmol/l (using cubic splines with 4 knots). **a**. Hazard ratio throughout the study period. **b**. Hazard ratio in the pre-ART period. *Multivariable analysis adjusted for age sex, CD4, Hb, BMI, oedema, TB treatment, Cotrimoxazole, CRP, grip strength, site, follow-up period and study arm. Results shown for baseline phosphate <3mmol/l

Risk of death before starting ART appears higher among patients with very high phosphate values (Fig. 1), however, there is no evidence of an association in the study overall (Table 3). There were few patients with extremely abnormal values (Table 2), so power to detect an association is low.

One fifth of patients died from confirmed or suspected TB. Respiratory tract infections and diarrhoea were also common diagnoses for patients who died. Cause of death was not confirmed for one third of patients, the majority of whom died at home (Additional file 1: Table S1).

## Discussion

The high mortality observed around the initiation of ART is a major concern for programme managers and clinicians in Africa. To our knowledge, this is one of the first studies to assess risk factors for mortality in both the period from referral for ART and in the early post-ART period among malnourished adults, a group comprising a third of new patients in some low-resource settings [2]. Well-established risk factors we found consistent with previous studies [4, 6, 9] include low BMI, low fat-free mass, low CD4, high CRP, and male sex. Less-frequently investigated factors associated with an increase in mortality include oedema, low grip strength and low MUAC. More surprising results were seen with TB treatment and phosphate level.

Overall mortality was high in our population but, when stratified by BMI (results not shown), was similar in the post-ART period to that found in a large Tanzanian study [3]. This suggests our patients were not unusual in their characteristics or medical care compared to other malnourished HIV-positive adults in Africa, and supports the validity of the extremely high mortality rates seen in the pre-ART period. Other studies also show higher mortality before, compared to the first months after, starting ART [1, 22]. When examined in more detail, the mortality rate in our study increased through the pre-ART period, highlighting the extreme vulnerability of these patients and further supporting the theory that much early ART mortality results from advanced disease. This confirms the value of immediate ART initiation, as recently recommended by WHO for all persons with HIV worldwide [23].

We expected receipt of TB treatment, as a proxy for TB infection, to be associated with higher risk of mortality [24] but it was actually strongly protective. This could suggest a substantial burden of undiagnosed TB is present in this population. A study conducted in Zambia and Tanzania at the same time as ours but among a population with median BMI of 19.3 found a similar proportion of patients (16 %) were on TB treatment at baseline [25]. However, previous work in Tanzania has shown that TB explains most of the weight deficit seen in TB/HIV co-infected patients [26] so we would expect to see more TB in our cohort of malnourished adults than in a better-nourished population. It is also possible that TB treatment was effective against other pathogens, including those causing diarrhoea [27], which might otherwise have contributed to mortality. Patients already on TB treatment when first assessed for ART may be better care-seekers and therefore healthier. However the protective effect of TB treatment on post-ART mortality remained when only studying patients who started TB treatment after recruitment. These findings call for greater efforts to be made to diagnose and treat TB, especially among malnourished patients for whom algorithms and investigations focused on respiratory symptoms and signs may be less sensitive. They may also support empiric treatment for TB, especially in undernourished individuals; however, prioritising TB treatment for HIV positive South Africans with high probability of TB, including BMI<18.5, did not reduce mortality [28].

Our prior studies, which formed the foundation for NUSTART’s design, found low pre-ART serum phosphate levels to be independently associated with early ART mortality [5, 29]. In contrast, in our population, serum phosphate did not show evidence of an association with overall mortality and high phosphate appeared to carry a greater risk in the pre-ART period. Our attempt to treat patients having low electrolyte levels with oral electrolyte supplementation may have reduced any impact of low phosphate on mortality. Serum phosphate levels are known to change over time and we are planning more detailed analysis of electrolytes as time-varying variables.

In this population, oedema carried a high risk of death. Very few patients presented with oedema, so the risk estimate lacked precision; however, oedema can be a sign of advanced malnutrition, so it is not a surprising finding. MUAC, which is routinely used in the diagnosis and monitoring of malnourished children but rarely for adults, was also strongly associated with mortality. It is inexpensive and simple to measure, even in very ill patients unable to stand, and may therefore have a role in resource-constrained settings and for severely ill patients where measuring BMI is not an option.

While some risk factors were significantly associated with mortality in both the pre-and post-ART periods, there are some interesting exceptions. Male sex was a risk only in the pre-ART period, possibly reflecting later care-seeking by men but an equivalently good response to ART as in women.

Grip strength is often considered a functional indicator of lean mass, and there is also evidence of an association with lipodystrophy and inflammation in HIV patients [30]. However, in this population low grip strength carried a risk for mortality independent of BMI and serum CRP. Grip strength of HIV positive adults starting ART in Ethiopia was improved by a lipid-based nutritional supplement [31] and it has been used as a component of ‘frailty’ in monitoring African patients on ART [32]. Therefore, further research is warranted into what affects grip strength, how it interacts with body composition, fat distribution, and inflammation, and how it performs as an adjunct to clinical care in populations at different stages of HIV or ART treatment.

Mortality was higher in Mwanza than Lusaka even in adjusted analyses that controlled for the slightly lower CD4 counts in Mwanza. This could be partly explained by the tracing of non-attendees to their home in Mwanza, but only by phone in Lusaka, therefore identifying more patients who died during the study. There may also have been additional, unmeasured factors indicative of more severe disease in Mwanza patients and the effect of localised conditions such as the higher incidence of malaria in Mwanza. Of note, once the Tanzanian patients were on ART treatment, they did as well as those in Zambia.

Our study benefited from a large sample size, excellent follow-up for the mortality outcome, and rich database of interview and anthropometric measures and biomarkers. It had several limitations as well. Due to financial constraints, we were unable to measure HIV viral load. Data on cause of death were often missing or unreliable, so we could not investigate how risk factors were associated with particular causes. Appetite could be analysed only for Mwanza patients because the appetite score was clustered and possibly unreliable in Lusaka, [18] so this analysis was under-powered. TB data relied on patient self-report which could be unreliable, however, this is unlikely to explain the large effect seen, especially in a population with high TB awareness and understanding. We could not analyse associations of particular ART regimens with mortality because there was a large difference in regimens between sites and the sites themselves differed in mortality risk.

## Conclusions

Our results extend knowledge of risk factors for mortality of HIV patients to the high risk period between referral for ART and ART initiation which in most African clinics lasts several weeks. The extremely high mortality that occurred during this period provides further support for minimising delays in diagnosis and treatment initiation, especially among malnourished patients. The strong association between TB treatment and reduced mortality supports more commitment to diagnosis of TB of underweight patients commencing ART.

## Additional file

Additional file 1: Table S1. Cause of death during the pre and post-ART periods. Cause of death obtained from hospital records for patients who died during the study, presented separately for deaths during the pre-ART and post-ART time periods. (DOCX 14 kb)

## Abbreviations

3TC: Lamivudine
ART: Anti-retroviral therapy
AZT: Zidovudine
BMI: Body Mass Index
CI: Confidence Interval
CRP: C-reactive protein
CV: Coefficient of variance
EFV: Efavirenz
FTC: Emtricitabine
HR: Hazard ratio
IQR: Inter-quartile range
LNS: Lipid-based nutritional supplement
LNS-VM: LNS with high level vitamins and minerals
MUAC: Mid-upper arm circumference
NIMR: National Institute for Medical Research (Tanzania)
NUSTART: Nutritional Support for African Adults Starting Antiretroviral Therapy
NVP: Nevirapine
PCA: Principal component analysis
QC: Quality control
SES: Socio-economic status
TB: Tuberculosis
TDF: Tenofovir
UTH: University Teaching Hospital (Zambia)
