# Supplementary material for: Risk factors for mortality among malnourished HIV-infected adults eligible for antiretroviral therapy
Source: BMC Infect Dis. 2016 Oct 12;16:562. doi: 10.1186/s12879-016-1894-3 (PMC5062813; doi:10.1186/s12879-016-1894-3)
Supplement: Additional file 1: Table S1. — Cause of death during the pre and post-ART periods. Cause of death obtained from hospital records for patients who died during the study, presented separately for deaths during the pre-ART and post-ART time periods. (DOCX 14 kb) [file 12879_2016_1894_MOESM1_ESM.docx]

**Supplementary Table 1: Cause of death during the pre and post-ART periods**

| Cause of death | Pre-ART n(%) | Post-ART n(%) | Total n(%) |
| --- | --- | --- | --- |
| Total | 151 | 195 | 346 |
|  |  |  |  |
| TB^1^ | 23 (15.2) | 46 (23.6) | 69 (19.9) |
| Malaria (presumptive or confirmed) | 3 (2.0) | 3 (1.5) | 6 (1.7 ) |
| Vomiting/diarrhoea | 15 (9.9) | 22 (11.3) | 37 (10.7) |
| Other infectious disease^2^ | 1 (0.7) | 2 (1.0) | 3 (0.9) |
|  |  |  |  |
| Cervical cancer | 2 (1.3) | 0 | 2 (0.6) |
| Kaposi sarcoma | 0 | 1 (0.5) | 1 (0.3) |
|  |  |  |  |
| Anaemia | 12 (8.0) | 14 (7.2) | 26 (7.5) |
|  |  |  |  |
| Meningitis/encephalitis^3^ | 9 (6.0) | 5 (2.6) | 14 (4.1) |
| Epilepsy | 1 (0.7) | 0 | 1 (0.3) |
|  |  |  |  |
| Diseases of the circulatory system^4^ | 1 (0.7) | 3 (1.5) | 4 (1.2) |
|  |  |  |  |
| Respiratory tract infections | 27 (17.9) | 18 (9.2) | 45 (13.0) |
| Asthma | 0 | 1 (0.5) | 1 (0.3) |
|  |  |  |  |
| Disorders of liver^5^ | 2 (1.3) | 3 (1.5) | 5 (1.5) |
|  |  |  |  |
| Diseases of urinary system^6^ | 1 (0.7) | 2 (1.0) | 3 (0.9) |
|  |  |  |  |
| Cause unknown | 50 (33.1) | 68 (34.9) | 118 (34.1) |

1 includes those with suspected TB as well as those started on treatment

2 includes sepsis, chicken pox, gangrene

3 includes bacterial, viral and cryptococcal

4 includes heart failure and stroke

5 includes hepatoma, drug induced hepatits and liver failure/jaundice

6 includes retention of urine/urethral stricture, orchitis (being investigated for testicular tumour) and renal failure
